# The UCSC Genome Browser database: 2019 update

**DOI:** 10.1093/nar/gky1095

**Published:** 2018-11-08

**Authors:** Maximilian Haeussler, Ann S Zweig, Cath Tyner, Matthew L Speir, Kate R Rosenbloom, Brian J Raney, Christopher M Lee, Brian T Lee, Angie S Hinrichs, Jairo Navarro Gonzalez, David Gibson, Mark Diekhans, Hiram Clawson, Jonathan Casper, Galt P Barber, David Haussler, Robert M Kuhn, W James Kent

**Affiliations:** 1 Genomics Institute, University of California Santa Cruz, Santa Cruz, CA 95064, USA; 2 Howard Hughes Medical Institute, University of California Santa Cruz, Santa Cruz, CA 95064, USA

## Abstract

The UCSC Genome Browser (https://genome.ucsc.edu) is a graphical viewer for exploring genome annotations. For almost two decades, the Browser has provided visualization tools for genetics and molecular biology and continues to add new data and features. This year, we added a new tool that lets users interactively arrange existing graphing tracks into new groups. Other software additions include new formats for chromosome interactions, a ChIP-Seq peak display for track hubs and improved support for HGVS. On the annotation side, we have added gnomAD, TCGA expression, RefSeq Functional elements, GTEx eQTLs, CRISPR Guides, SNPpedia and created a 30-way primate alignment on the human genome. Nine assemblies now have RefSeq-mapped gene models.

## INTRODUCTION

The University of California at Santa Cruz (UCSC) Genome Browser (1) is a viewer for genome annotations, primarily those from human and mouse genomes. The UCSC Genome Browser team has continually added data and software features to the website since 2001 and currently hosts 195 assemblies and 105 species (Menu: ‘Genomes > Other Genomes’). We continue to add new species and assemblies, but most of our user base focuses on human and mouse. As a result, most of our annotation is concentrated on these two species.

Apart from showing the DNA sequence itself, the Genome Browser is primarily used to display genome annotations. Most of these are derived in some way from aligning sequences to the genome, created by us or other groups, and they are stored and shown as small rectangles with defined chromosome:start-end coordinates (i.e. chr10:123 400–123 499) or graphs of annotation density. Annotations are organized into tracks, each with a name, description and configuration options. A track can be set to one of the four display modes, from hidden, to a single line (‘dense’), thin lines (‘squish’), normal lines (‘pack’), up to ‘full’ with one annotation per line (see our new video https://youtu.be/jKix2B3hwnw). The most-used tracks on the human genome are the various gene models, followed by variants and ENCODE annotations. Documentation for each track is available by right-clicking it, clicking on its name below the image or the vertical gray bar next to the track. In addition, files in our public ‘makeDb/doc’ directory contain the UNIX commands to build any track (see http://bit.ly/makeDocs). At the top of the documentation page, tracks can be configured. Depending on the track type, one can change the size, show only certain features, activate coding sequence codon display, etc. To share a complete track selection and configuration, the user can create a stable link using ‘My Data > My Sessions’ and share with collaborators or further create a ‘Public Session’ hosted on our servers.

In addition to the main Genome Browser, the website contains related utilities in the ‘Tools’ menu. These utilities may find sequences in the genome (BLAT, in-silico PCR), add annotations to uploaded variants (Variant Annotation Integrator), let users inspect, join and download our annotations as text files in various table formats (Table Browser, Data Integrator) or map annotations between genomes (LiftOver and also ‘View > In Other Genomes’). Other tools show data not directly located on chromosomes, like VisiGene for in-situ images and our viewer for gene/protein interaction networks (‘Tools > Gene Interactions’).

All genome annotations can also be downloaded as text files (‘Downloads > Genome Data’) or retrieved by MySQL (‘Downloads > MySQL Access’). Under ‘Downloads > Utilities’, we provide several hundred tools that allow users to convert and analyze our genomics data files on a UNIX-like operating system. Finally, we provide all Genome Browser source code along with scripts that help install these or a Genome Browser with selected assemblies on the user's own server and a completely configured virtual machine (‘Mirrors > Mirroring Instructions’).

A major aim of the group over the last few years has been the development of new features that allow researchers to show their own annotations on the Genome Browser. Under ‘My Data > Custom Tracks’ a user can type in custom annotations (e.g. ‘chr1 1 1000 my_annotation’) or paste URLs of next-generation sequencing files, such as BAM, VCF and many other formats. A research group can use ‘Track Hubs’ (2), to create a group of customized tracks, configure and document them in detail and link to it from a research article. Authors of track hubs can contact us at genome-www@soe.ucsc.edu to have their hub added to the list of public track hubs. At the moment around 60 groups have done so, with 11 hubs added in 2018 (see Supplemental Table S1), and a broad selection of tracks is available as a result. Examples include cis-regulatory annotations created by JASPAR (3), ReMap (4), the Eukaryotic Promoter Database (5), ENCODE(6), the Ensembl regulatory build (7) and Brain and Roadmap Epigenomics (8), RNAs from FANTOM5 (9), Mouse Alleles from MGI (10), lncRNAs from LNCipedia (11), Cancer genomics (showing variants from TCGA (12)) and many others. Most of the tracks were created for the human genome, but there are also *Caenorhabditis elegans* WormBase alignments and DNA methylation levels in pigs. Researchers can even submit annotations for genomes we do not host by adding the genome sequence to create an ‘Assembly hub’. More information is available at https://genome.ucsc.edu/goldenpath/help/hgTrackHubHelp.html

## NEW ASSEMBLIES AND ANNOTATIONS

A detailed list of the annotation tracks that were added this year is available in Supplemental Table S2.

### Genomes from more organisms and the CHO cell line

In the past year, seven new assemblies, three for new species, were added. The new organisms are African clawed frog (xenLae2), Lamprey germline (petMar3) and a new assembly, the Chinese Hamster ovary cell line CHO-K1 (criGriCHOV1). Existing genomes that were updated are *Xenopus tropicalis (xenTro9)*, pig (susScr11), chimp (panPan2) and zebrafish (danRer11).

### Genotype-Tissue expression project

The GTEx Gene track on human genome assemblies displays expression levels of genes in 53 different tissue types (13). We have augmented this annotation with a transcript-level expression track, GTEx Transcript. We have also added GTEx tracks representing eQTLs, genetic variants associated with and likely causal for the differences in gene expression (14). Two new tracks (Combined eQTL and Tissue eQTL) show gene expression quantitative trait loci within 1 Mb of gene transcription start sites (cis-eQTLs), significantly associated with gene expression and in the credible set of variants (by CAVIAR analysis) for the gene at a high confidence level (95%). More distant SNP/Gene interactions are in a new separate track, GTEx Trans eQTLs, which is hosted on the public GTEx Analysis track hub at UCSC. More details for these tracks are at https://genome.ucsc.edu/cgi-bin/hgGtexTrackSettings?db=hg19&g=gtexEqtlCluster and http://hgdownload.soe.ucsc.edu/hubs/gtexAnalysis/gtexTransEqtl.html. Finally, the animated GTEx Body Map graphic has been deployed to a stand-alone web page, freely available for linking or image capture: https://genome.ucsc.edu/gtexBodyMap.html.

### New NCBI RefSeq tracks

RefSeq (15) is a group at NCBI that manually curates transcript sequences. To obtain genome annotations, these sequences have to be aligned to the genome and can sometimes differ from it. The UCSC Genome Browser has always provided a BLAT-based mapping (16) and NCBI made available genome annotations which differ slightly. For this reason, we created the ‘NCBI RefSeq’ track from these original NCBI transcript mappings for hg38/GRCh38, which are released every two months. This year we have added this track to eleven model organism assemblies: mouse, *C. elegans*, yeast, zebrafish, fruitfly, *X. tropicalis* and rat. Our new ‘NCBI RefSeq’ tracks still include a subtrack with transcript mappings performed by UCSC, both for backwards compatibility and to allow comparison between the NCBI and UCSC mappings, which may be informative especially when they differ. In addition, the UCSC tracks are continually updated while the NCBI RefSeq tracks are only released every few months but as a stable and archived ‘NCBI Annotation Release’. At the time of writing the current annotation release is 109. It should not be confused with the ‘Refseq Release’ which contains only the sequences and is currently at release 89. Since the databases for fruit fly, yeast and C. elegans (FlyBase, SGD and WormBase, respectively) submit to RefSeq, our gene models on these organisms now match the main model database. More information on these NCBI alignment tracks can be found at http://genome.ucsc.edu/blog/the-new-ncbi-refseq-tracks-and-you.

### ‘RefSeq functional elements’

The RefSeq group now also curates functional elements in non-genic regions (17). These include experimentally-verified gene regulatory regions, known structural elements, well-characterized DNA replication origins, and clinically-significant sites of DNA recombination and genomic instability. We have added them as tracks on the human hg38/GRCh38 and mouse mm10/GRCm38 assemblies.

### Ensembl/gencode updates

Ensembl gene models are built through an automated pipeline and they are defined through genome coordinates, so they do not have to be mapped to a genome (19). Last year, 59 of our assemblies were updated to Ensembl 91. GENCODE (18) is a project that manually curates gene transcript models. It differs from RefSeq in that it produces a larger set of transcripts, as it has slightly different curation criteria, and the transcripts are defined through genome coordinates. Ensembl uses the GENCODE transcripts for human and mouse instead of using the automatic pipeline. In the UCSC Genome Browser, GENCODE/Ensembl is the default gene track for hg38 and mm10. We added tracks for GENCODE V27 (equivalent to Ensembl 90/91) and V28 (equivalent to Ensembl 92/93) to hg38, both of which are now also available on hg19 as backmaps via liftOver from hg38.

### ‘Cancer gene expression’

We have added a track showing RNA expression data from The Cancer Genome Atlas (TCGA). Our track is based on a recent re-alignment to hg38 for 33 types of cancer (20).

### ‘gnomAD’

The successor to the exome-only database ExAC (21), gnomAD is a collection of variants found in whole genome and exome sequencing studies. It consists of two callsets: variants from 123 136 exomes and 15 496 whole genomes of unrelated individuals. They were sequenced as part of various population-genetic and disease-specific studies collected by the Genome Aggregation Database (gnomAD), release 2.0.2. The track is available on hg19.

### ‘SNPedia’

SNPedia (22) is a wiki with descriptions of human variants. While the website has 107k pages, only ∼5000 contain manually typed text. We have added a track with all possible pages and another one for just the pages with typed text, for both hg19 and hg38.

### More alignments

We have added a ‘mostly-primate’ 30-way alignment track for the human hg38 assembly. In includes 27 primate genomes and three outgroups: mouse, human and armadillo. We have also added a new 11-species Multiz Conservation track for frog (xenTro9) that includes African clawed frog, Tibetan frog, Painted turtle, Opossum, Chicken, Lizard, Dog, Human, Mouse and Fugu.

## SOFTWARE IMPROVEMENTS

### Improved search

For improved compatibility with other genome browsers, our position/search box now accepts chromosome names in non-UCSC formats, e.g. a GenBank/INSDC or RefSeq accession or just a single number. For example, where users previously had to type ‘chr1’, they can now enter ‘NC_000001.11’ (RefSeq), ‘CM000663.2’ (GenBank) or just ‘1’. Since last year, the same search box also accepts Human Genome Variation Society (HGVS) variant nomenclature (23). Examples are ‘NM_005915.5:c.1917+326C>T’ or ‘NP_004324.2:p.V600A’. We also support a less official but more commonly used description that just includes the gene symbol, the amino acid (optional) and the position, e.g. ‘BRAF V600’. The HGVS feature has now been expanded to variants located on Ensembl/GENCODE transcripts and on LRG genomic and protein sequences, e.g. ‘ENST00000002596.5:c.-108-6848A>G’ or ‘LRG_456p1:p.Ser190Leu’. More information on other supported search terms in our position box is available at https://genome.ucsc.edu/goldenpath/help/query.html.

### Track collections

This year we have added an entirely new tool, the Track Collection Builder.

**Figure 1.**
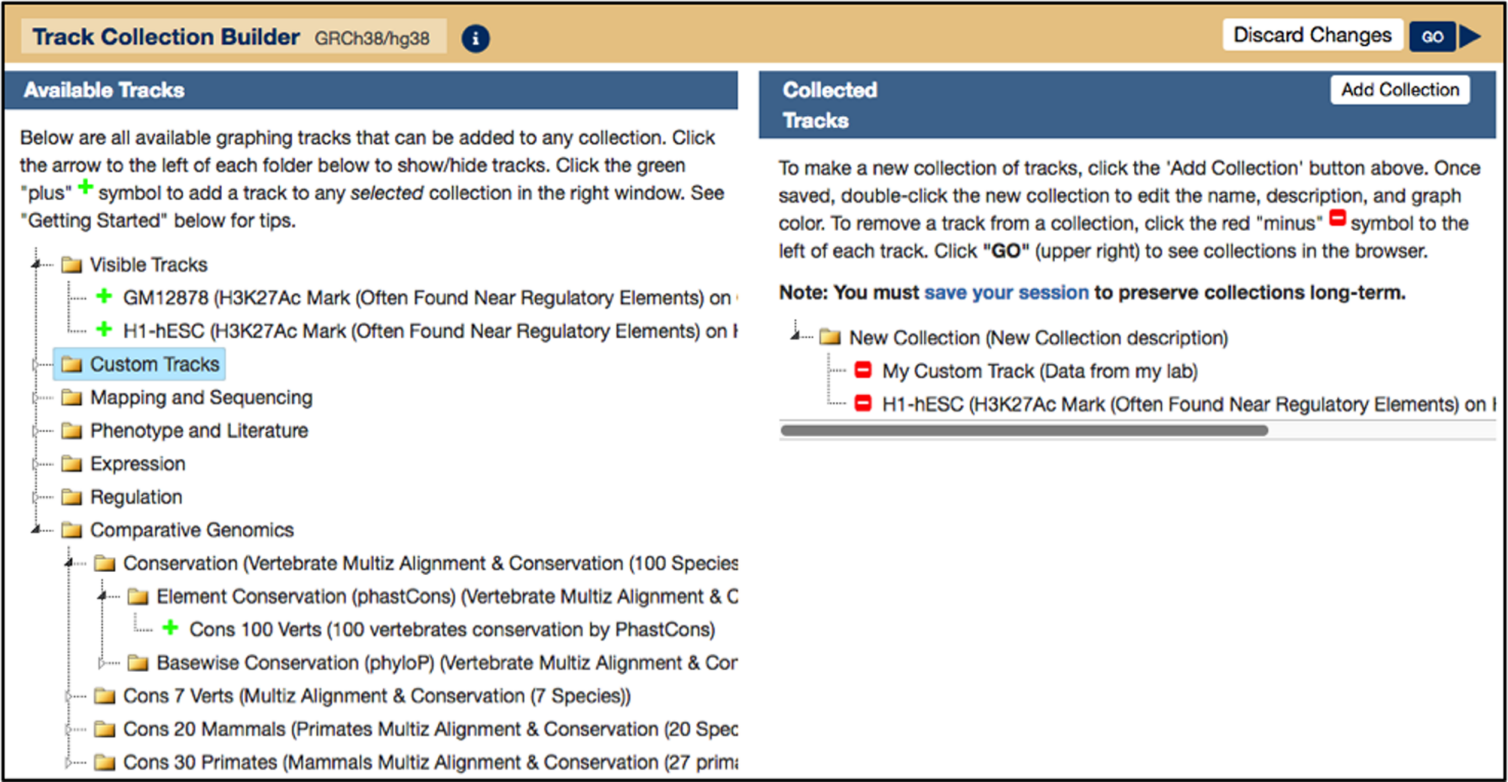
The Track Collection Builder tool. The left pane, ‘Available Tracks’ displays tracks that are available to add to a new collection. The first folder, ‘Visible Tracks’ displays all supported tracks that are currently visible in the main Browser image. Below that are expandable folders which show selectable tracks from all other Browser groups, regardless of Browser visibility. To add custom tracks or tracks from hubs, first load them into the Browser and then add them to a collection. This creates a ‘clone’ of the existing tracks and adds them to the new collection. The right pane, ‘Collected Tracks’ displays current collections. This New Collection consists of data from ‘My Custom Track’ and a native H1-hESC track. The ‘GO’ button in the top right corner allows the user to view the collection in the Browser.

The tool allows multiple existing graph tracks to be copied and grouped together into one collection. Custom tracks and tracks from hubs can be included in collections alongside native tracks produced at UCSC. More tracks can also be added to existing collections through the right-click menu. Once grouped, settings (e.g. y-axis auto-scaling, track height, graph color, transparent overlay) can be applied to all tracks at once. All tracks in a collection can be sorted by similarity or magnitude, and they can be added or subtracted from one another, as shown on Figure 1. Supported data types are limited to those that display continuous graphing data (wiggle, bigWig, and bedGraph). As with all new Browser tools, the Track Collection Builder comes with a detailed help page (https://genome.ucsc.edu/cgi-bin/hgCollection#INFO_SECTION) and a companion getting-started video (https://youtu.be/2_RiIY07omY).

### New track type – bigNarrowPeak

For ChIP-Seq peaks of the ENCODE project we introduced the narrowPeak format, a BED-like custom track text file format that stores the signal peak, its summit, the *P*-value and the *q*-value (24). A binary version of this format is now available for use in custom tracks and track hubs—the format is an extension of the existing bigBed binary format. The ENCODE DCC Portal creates (6) tracks of this type. It is available under ‘My Data > Track Hubs’. More information on this format can be found at https://genome.ucsc.edu/goldenPath/help/bigNarrowPeak.html.

### New track type—interact and bigInteract

**Figure 2.**
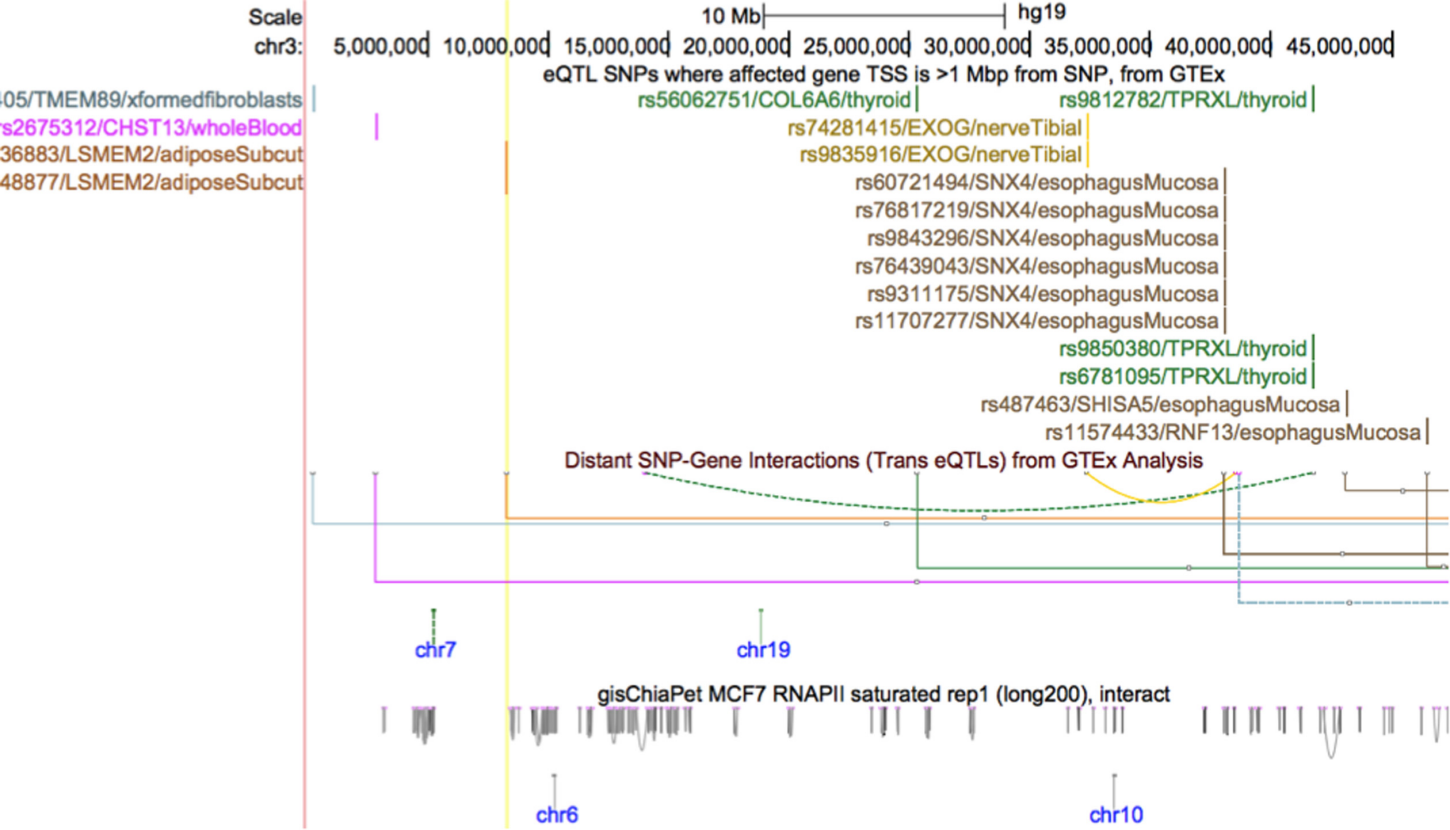
An example of the new interaction track format: shown are the eQTL SNP track and two interaction type tracks on chromosome 3. The topmost interaction track shows distant SNP/Gene interactions identified by GTEx analysis, colored by tissue type using GTEx conventions. Mouseover and click-through provide information including interaction size and strength, as well as identifying the endpoint SNP and gene. The companion track labels the interaction SNP’s with rsID, affected gene and tissue type (rs56062751/COL6A/thyroid). Interactions with both endpoints in the viewing window are displayed as curves—those with an endpoint outside the window are rectangular. The track on the bottom shows chromatin interactions, identified by ChIA-PET in the MCF-7 cell line, gray-scaled based on strength of interaction.

For chromatin interaction data we previously added support for the longRange/longTabix format, which was developed at Washington University in St. Louis and is also supported by Ensembl. To provide a curve-based display and support colors, scores, and names for the endpoints and interactions, we have now developed the new interact/bigInteract format (Figure 2). This new track type provides better representation and display of low-density chromatin interaction data such as ChIA-PET as well as functional interaction data such as SNP/gene effects (e.g. eQTLs). The track format supports both inter- and intra-chromosomal interactions. Inter-chromosomal relationships are displayed as a vertical tick mark (labeled if space permits, with the other chromosome name), in a horizontal band below the intra-chromosomal interactions. Intra-chromosomal interactions with both endpoints in view are represented by curves that stretch between those ends. When one or both endpoints are outside the view window, those interactions are displayed by straight lines stretching to the corresponding edge of the view window. Interaction tracks can be configured by the user to change the track height, display mode (curve, ellipse or rectangle), and can be filtered by score (interaction strength). Instructions are available at https://genome.ucsc.edu/goldenPath/help/interact.html.

### Command line tools

Almost all the functionality on our web interface is also available through UNIX command line tools. For example, variants can be mapped to transcripts with *vcfToHgvs and* annotated with *vai.pl*, while bigBed files can be inspected with *bigBedToBed*. A recent blog post demonstrates how to use a few of these tools (http://bit.ly/kentTools). The easiest way to install the tools is to download the pre-compiled binaries (‘Downloads > Utilities’). Third party groups have started to make our tools available through Bioconda (25) (e.g. ‘conda install ucsc-bigbedtobed’) and Docker (‘ucscuserapps’). Changes to our source are visible on our GitHub repository https://github.com/ucscGenomeBrowser/kent. Our preconfigured virtual machine (GBiB) has an option to add these tools. Our source code, the command line tools, an installation program (GBiC) and the virtual machine are all available https://genome-store.ucsc.edu (commercial users must obtain a license). Most parsers and format converters useful for other projects do not require a license and are in the public domain, see https://genome.ucsc.edu/license/.

## OUTREACH AND CONTACT INFORMATION

In the last year the Genome Browser's training team provided >20 seminars and workshops to help users take advantage of the latest features. Outreach is also supported by regular updates to the training documentation (https://genome.ucsc.edu/training/) and blog (http://genome.ucsc.edu/blog/) with videos and in-depth descriptions of new Browser features. The training documentation also includes information on how to submit a request for a workshop.

General contact information for the UCSC Genome Browser can be found on the website at https://genome.ucsc.edu/contacts.html, including information for accessing our email support list and the archive of previously answered mailing list questions. UCSC also maintains mirrors in Germany and Japan with the gracious assistance of the University of Bielefeld, Germany and the RIKEN Institute of Japan, respectively. Those sites can be found at https://genome-euro.ucsc.edu and https://genome-asia.ucsc.edu.

## PLANS FOR THE FUTURE

The UCSC Genome Browser team has several features in mind for the coming year. For VCF and possibly bigBed tracks a new lollipop display will show the allele frequency of variants with vertical bars. We intend to make it easier to search a single sequence against multiple assemblies in BLAT. Finally, some features of the Genome Browser that required command line tools will be made available through a RESTful API, e.g. sequence retrieval and annotations within a range. We will also add a tool for the exploration of single cell RNA-seq datasets.

## Supplementary Material

Supplementary Data
