# Supplementary material for: The UCSC Genome Browser database: 2019 update
Source: Nucleic Acids Res. 2018 Nov 8;47(Database issue):D853–8. doi: 10.1093/nar/gky1095 (PMC6323953; doi:10.1093/nar/gky1095)
Supplement: Supplementary Data [file gky1095_supplemental_files.pdf]

Supplementary Table 1. Track and assembly hubs added to UCSC's Public Hubs listing in the last year

Track Hub Name	Provider	Assemblies
Human cellular microRNAome barCharts	Marc Halushka & Arun Patil: Johns Hopkins University School of Medicine	hg38
JASPAR 2018 TFBS	David Arenillas & Robin van der Lee:<br>JASPAR Database	hg38, hg19, ce10, dm6, sacCer3, danRer10, mm10
Zebrafish Zygotic Genome ActiviationTranscripts Hub	Icahn School of Medicine at Mount Sinai, New York	danRer10
IDEAS roadmap 20states	Yu Zhang & Ross Hardison: Pennsylvania State University	hg19
ReMap Regulatory Atlas	Benoît Ballester & Jeanne Cheneby: Aix-Marseille University - Technological Advances for Genomics and Clinics (TAGC)	hg38
MGI Alleles and Phenotypes	Paul Hale: Mouse Genome Informatics	mm10
Seq-data on mm9 NS5 cells	Juan Luis Mateo Cerdán: University of Oviedo	mm9
LNCipedia 5.0	Pieter-Jan Volders: Center for Medical Genetics Ghent (CMGG) - Computational Omics and Systems Biology Group (CompOmics)	hg38, hg19
Brain Epigenome Hub	Peter Hickey: Johns Hopkins Bloomberg School of Public Health	hg19
Fetal retinal pigment epithelium (RPE) and iPSC-derived RPE track hub	Frazer lab: UC San Diego	hg19
dbRIP: Database of Polymorphic and Species-specific Retrotransposons in Humans	Brock University, St. Catharines, Ontario, Canada	hg38. hg19

Supplementary Table 2. Annotation tracks added or updated within the last year.

KEY: N = new, NU = new & updated, U = updated, AU = automatically updated

**Track Name**	**New/Update Status**	**Human assemblies**	**Mouse assemblies**	**Other assemblies**
TCGA barChart transcript and gene tracks	N	hg38		
GENCODE Genes V28	N	hg38		
30-species (27 mammals) Conservation	N	hg38		
Mappability track (from Hoffman Lab Mappability Hub)	N	hg38		
SNPedia	N	hg38, hg19		
GRCh37 Patch 13	N	hg19		
Genome Aggregation Database (gnomAD) variants	N	hg19		
DECIPHER sequence variant	N	hg19		
GTEx Trans eQTL	N	hg19		
GTEx Expression QTL	N	hg19		
GENCODE Genes V28 (lifted from hg38)	N	hg19		
NCBI RefSeq Functional Elements	N	hg38	mm10	
NCBI RefSeq Gene	N	hg38, hg19	mm10	ce11, sacCer3, danRer10, danRer11, dm6, xenTro7, xenTro9, rn6
UCSC Genes	N		mm10	
GENCODE Genes VM16	N		mm10	

CRISPR with 10K shoulders	N		mm10	
CRISPR	N			ce11, CHO K1 cell line
11-species Conservation	N			xenTro9
Chains & Nets (for many assemblies)	N	various	various	various
Ensembl Genes v90, v91, v92	N	various	various	various
Human Gene Mutation Database (HGMD) Public Variants	U	hg38, hg19		
ClinVar Nucleotide Variants	U	hg38, hg19		
GenBank Updates (RefSeq Genes, ESTs, RNAs)	AU	Most assemblies updated	Most assemblies updated	Most assemblies updated
GRC Incident	AU	hg38, hg19	mm10, mm9	danRer10, danRer7, galGal5
Gene Reviews	AU	hg38, hg19, hg18		
NHGRI Catalog of Published GWAS	AU	hg38, hg19, hg18		
OMIM Genes & Phenotypes	AU	hg38, hg19, hg18		
ClinGen Research	AU	hg38, hg19		
ClinVar Variants	AU	hg38, hg19		
DECIPHER	AU	hg19		
LOVD	AU	hg19		

Supplementary Table 2. Annotation tracks added or updated within the last year.
